# Familial Hyperparathyroidism: A Diagnostic and Treatment Challenge in Saudi Arabia

**DOI:** 10.7759/cureus.28434

**Published:** 2022-08-26

**Authors:** Hind AlNassar, Mahmoud Machmouchi, Ashraf alnosair

**Affiliations:** 1 General Surgery, Almoosa Specialist Hospital, Al Ahsa, SAU; 2 Pediatric Surgery, Almoosa Specialist Hospital, Al Ahsa, SAU

**Keywords:** hypercalcemia, multiple endocrine neoplasia type 1, parathyroid adenoma, hyperparathyroidism, familial hyperparathyroidism

## Abstract

Familial hyperparathyroidism is a rare, inherited endocrine disorder characterized by abnormally elevated serum calcium due to increased parathyroid hormone levels.

In this case report, we present a two-day-old male newborn who was admitted with severe respiratory distress, hyperparathyroidism, and hypercalcemia with a family history of hyperparathyroidism in his two siblings, both diagnosed in childhood and treated with parathyroidectomy. He was diagnosed with familial hyperparathyroidism without other endocrinopathies. His left parathyroid glands were surgically removed, and post-operatively, his parathyroid hormones and calcium levels normalized. Pathological examination of the removed parathyroid glands confirmed parathyroid hyperplasia.

This is a successfully managed case of familial hyperparathyroidism in the neonatal period. Therefore, as the patient grows up, a close follow-up is recommended for early detection and managing multiple endocrine neoplasia type 1 that may be present later in life.

## Introduction

Familial hyperparathyroidism (FHPT) is a rare inherited endocrine disorder, prevalent in 2-5% of primary hyperparathyroidism cases [1,2]. It usually presents at the same age as multiple endocrine neoplasia (MEN) type 1 and type 2A with high parathyroid hormones, parathyroid tumors, and hypocalciuric hypercalcemia [3]. Apart from MEN, it is also associated with other disorders, such as hyperparathyroidism-jaw tumor syndrome, severe neonatal hyperparathyroidism, and autosomal dominant moderate hyperparathyroidism [1,4]. Most patients with FHPT also carry a germline genetic mutation causing parathyroid tumors [5]. Since the parathyroid hormones are involved in calcium regulation by responding to the calcium-sensing receptor (CASR), the germline mutation of CASR leads to hypercalcemia and excessive parathyroid hormones [1,5]. Thus, FHPT patients have symptoms of resulting hypercalcemia, hyperparathyroidism, hypophosphatemia, and other related imbalances. The symptoms include fatigue, lethargy, concentration and memory impairments, constipation or bloating, polyuria, nephrolithiasis, osteoporosis, and other skeletal abnormalities when it’s severe. In the presence of endocrine tumors, patients can have compression-related symptoms [5]. Familial hyperparathyroidism is tricky to diagnose, as ruling out other endocrinopathies is necessary and its management is mostly surgical. In this case report, we present a neonate diagnosed with familial hyperparathyroidism without any other endocrinopathies.

## Case presentation

**Figure 1:**
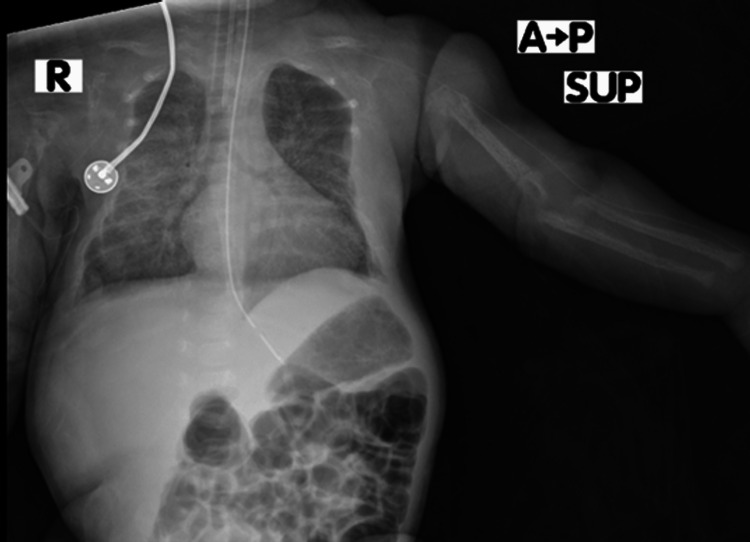
Chest x-ray showing a bell-shaped chest

A two-day-old male term neonate developed respiratory distress after birth with desaturation of oxygen saturation (SpO2) to 80% on mechanical ventilation after intubation and an absence of meconium within the first 24 hours. The neonate was born by cesarean section (CS) due to a previous CS scar to a healthy gravida 3, para 2 mother, with an uneventful antenatal period. His birth weight was 2550 grams with normal umbilical cord blood gas analysis. His brother and sister were diagnosed with hyperparathyroidism at the age of 40 days and 4 months, respectively. Both siblings underwent total parathyroidectomy at the age of 2 and 7 months, respectively. On examination, he was stable and afebrile with SpO2 of 90%, a respiratory rate of 65/minute, and a heart rate of 165 beats/minute. On the ventilator, the assist-control was 22/5 and the fraction of inspired oxygen (FiO2) was 40%. Other systemic examination findings were normal. At the age of 15 days, hyperparathyroidism was suspected based on the family history and the requested laboratory tests came back showing high total and ionized calcium and parathyroid hormone levels (Table 1). A pediatric endocrinologist advised ensuring good hydration, giving him Lasix three times a day, and monitoring thyroid-stimulating hormone (TSH) and T4 to rule out MEN syndrome. TSH and T4 monitoring showed a decreasing pattern as shown in Table 1. The genome sequence was requested, but it was not done due to a lack of insurance support. A chest X-ray showed a bell-shaped chest and widely separated ribs (Figure 1).

**Table 1:** Pre and postoperative laboratory investigations, TSH and T4 levels TSH: thyroid-stimulating hormone

Lab tests	Pre-operative	Post-operative (day 2)	Post-operative (day 4)
Parathyroid hormone (1.59 – 7.24 pmol/l)	376.96	77.74	
Total calcium (2.20 -2.70ml/dl)	>6ml/dl		
Ionized calcium (1.15 – 1.33 ml/dl)	2.15ml/dl	1.66	0.83
Thyroid stimulation hormones (TSH) and T4 monitoring			
Date	5/7/2020	7/7/2020	11/7/2020
TSH (<30mIU/L)	4.65 mlU/L	0.5 mlU/L	0.45 mlU/L
T4 (13.97 – 39.89 pmol/l))	137.73	10.27	15.8

Pediatric surgery was consulted and recommended to do neck magnetic resonance imaging (MRI) with a radioisotope but due to the financial constraints of the parents, it was not done. A simple ultrasound neck was performed which showed hyperplasia of the glands. The surgery was performed on day 17 of life, and intraoperatively, they found two nodules of thyroid tissue, which were removed and sent for pathological examination, which confirmed them to be of thyroid origin. The surgeon found the four parathyroid glands in their normal anatomical places with no nodules. Subtotal parathyroidectomy was done and sent for pathology. The pathological diagnosis later came back as parathyroid hyperplasia. Intraoperative parathyroid hormone monitoring showed an instant decrease from 376.96 pmol/l preoperative levels to 77.47 pmol/l 10 minutes after parathyroidectomy. Postoperatively, parathyroid hormone and calcium levels started to normalize on day 2 after surgery. He was discharged in stable condition after recovery on calcium gluconate 10% supplement for his low ionized calcium (0.83 ml/dl). Two weeks postoperatively, the patient was seen in the outpatient clinic doing fine. Parathyroid hormones and calcium levels were back within normal ranges.

## Discussion

We presented a case of familial hyperparathyroidism in a newborn who had a successful parathyroidectomy, after which the parathyroid hormones and calcium levels returned to normal and the symptoms resolved. In this case, pathological examination results showed parathyroid hyperplasia. Almost all cases of MEN type 1 have parathyroid hyperplasia, but they also complicate pituitary, thyroid, adrenal gland, and pancreatic endocrinopathies [3]. Our newborn patient had no other endocrinopathies. However, since parathyroid hyperplasia is the first sign of MEN type 1 and was discovered early in life for our patient, a long-term follow-up for possible development of other endocrinopathies is recommended [4,6].

Despite the parents' economic barriers preventing us from doing a genetic test, the family history of all siblings affected indicates a possible genetic cause, especially autosomal dominance, which is consistent with familial hyperparathyroidism, which is believed to be autosomal dominant [7]. The precise causative gene is unknown, but MEN type 1, CASR, and CDC73 (cell division cycle 73) gene mutations have been linked to the disease [8]. As a result, before testing for gene mutations, the first step in diagnosing familiar hyperparathyroidism is to rule out MEN type 1-related endocrinopathies and features of hyperparathyroidism-jaw tumor syndrome [1,5]. Though it was not done for our patient, MRI can show a low signal intensity mass on T1-weighted images and intermediate or high signal intensity on T2-weighted images in the case of ectopic parathyroid adenomas [9]. The chest X-ray of our patient showed a bell-shaped chest and wider rib separation similar to another previous case of neonatal hyperparathyroidism, indicating that hyperparathyroidism differential diagnosis should be considered in the case of a bell-shaped chest [10]. The bell-shaped chest was also reported in many other similar cases [11,12]. In addition, a bell-shaped chest also happens in respiratory distress cases similar to ours and in other bony dysplasia cases. Hyperparathyroidism causes bony resorption by favoring more catabolic effects on bones than anabolic effects [13], which is why it should be treated earlier to present skeletal abnormalities. The surgical management is parathyroidectomy and usually, intraoperative findings are; single adenoma or asymmetrical multiple parathyroid adenomas [1], which align with the intraoperative findings of two left superior and inferior parathyroid adenomas in our case with one right parathyroid gland adenoma.

## Conclusions

Our case demonstrated a typical case of familial hyperparathyroidism in neonates that were successfully managed by total parathyroidectomy. We recommend a close follow-up. The genetic test should also be done, and advocacy is needed for them and other patients in similar situations to be supported in order to afford all necessary care costs.
